# The Abbott TDx evaluated for T-uptake

**DOI:** 10.1155/S1463924684000432

**Published:** 1984

**Authors:** G. A. Harff

**Affiliations:** 1 Department of Clinical Chemistry Academic Hospital Free University De Boelelaan 1117 Amsterdam 1081 HV The Netherlands; 2 Department of Clinical Chemistry Academic Hospital Rijnsburgerweg 10 Leiden 2333 AA The Netherlands


Journal of Automatic Chemistry, Volume 6, Number 4 (October–December 1984), pages 214–215

### The Abbott TDx evaluated for T-uptake

### G. A. Harff*

*Department of Clinical Chemistry, Academic Hospital, Free University, De Boelelaan 1117, 1081 HV Amsterdam, The Netherlands*

### Introduction

The Abbott TDx therapeutic drug-monitoring system is based on fluorescence polarization. Polarization fluoroimmunoassay makes use of competitive binding, measuring the tracer binding directly, without the need for separation procedures. The attraction of non-radioactive polarization fluoroimmunoassay for the determination of T-uptake is that there is no radiation hazard and that the homogeneous method is very convenient.

### Materials

*Apparatus:* TDx Therapeutic Drug Monitoring System (Abbott Laboratories, Diagnostics Division, North Chicago, Illinois, USA).
*Reagents*: T-uptake reagent pack, Lot No. 55-167-HC (Abbott Laboratories). T3-uptake, $^{125}$I RIA kit, (Diagnostic Products Corporation, Los Angeles, California, USA).
*Specimen*: T-uptake calibrators No. 8100/9117-01, Lot No. CLI (Abbott Laboratories).
*Controls*: RIATRAC radioassay controls: RIATRAC 1, Lot No. HL 4001; RIATRAC 2, Lot No. HL 4002; and RIATRAC 3, Lot No. HL 4003 (Becton Dickinson Immunodiagnostics, Orangeburg, New York, USA).
Fresh patients' sera from the Academic Hospital were used for the method comparison.

### Results and discussion

### Replication experiment

Three replication experiments—'mini', 'midi', and 'maxi'—are described by the National Committee for Clinical Laboratory Standards (NCCLS) in the proposed standard PSEP-3 [1] and these were consulted for the evaluation. The experimental design permits estimation of within-run, between-run within a day, between-run between days, and total variance. The midi and maxi experiments permit calculation of the variance excluding and including carry-over effects. The TDx therapeutic drug monitoring system measures by fluorescence polarization immunoassay. An estimation of the effects of carry-over with the TDx was considered important—it is a discrete system and the probe may contribute to carry-over. Abbott Laboratories, in their *Operator's Manual* [2], claim that carry-over is less than 1·5%. The midi experiment is an efficient method where three different levels control materials need to be analysed for T-uptake six times per analytical run, with two runs per day for 20 working days. This gave a total of 240 observations at each level.

* Present address: Department of Clinical Chemistry, Academic Hospital, Rijnsburgerweg 10, 2333 AA Leiden, The Netherlands.

*Table 1. Statistical analysis of data from the replication experiment.*

| Level<br>Imprecision | Low<br>CV (%) | Mid<br>CV (%) | High<br>CV (%) |
|---|---|---|---|
| Within-run | 3·37 | 2·31 | 2·53† |
| | 3·87 | 1·84 | 1·89‡ |
| Between-run | 3·43 | 1·54 | 2·83† |
| (within a day) | 2·79 | 1·25 | 1·27‡ |
| Between-run | 2·62 | 1·34 | 2·10† |
| (between days) | 2·35 | 1·25 | 1·80‡ |
| Total | 4·45 | 2·52 | 3·31† |
| | 4·35 | 2·27 | 2·50‡ |

*Note*: mean concentration for the controls were 0·410 (low), 0·729 (mid) and 1·032 (high).
† Included carry-over.
‡ Excluded carry-over.

*Table 2. Within-run imprecision estimated from duplicate analyses of patients' samples.*

| *N* | Uptake (%) | Relative uptake† | CV (%) |
|---|---|---|---|
| T3-uptake (RIA) | | | |
| 33 | 24·66 | 0·87 | 3·71 |
| 33 | 29·22 | 1·08 | 3·96 |
| 34 | 34·62 | 1·38 | 4·48 |
| Total 100 | 29·55 | 1·11 | 4·26 |
| T-uptake (TDx) | | | |
| 33 | 27·07 | 1·13 | 2·31 |
| 33 | 30·26 | 0·97 | 2·25 |
| 34 | 35·87 | 0·78 | 2·27 |
| Total 100 | 31·12 | 0·96 | 2·31 |

† T-uptake values expressed in unitless numbers which represent ratios relative to a normal value.

Three levels of RIATRAC radioassay controls were used to perform the midi replication experiment. The results are given in table 1. An estimate of the percentage carry-over is obtained from the sequence of observations of levels: High 1, High 2, Low 1, Low 2 and Low 1, Low 2, High 1, High 2. H2 and L2 were assumed to be unaffected by carry-over because they are preceded by samples of the same concentration. A percentage carry-over is estimated by respectively $p = (L1-L2)/(H2-L2) \times 100\%$ and $p = (H1-H2)/(H2-L2) \times 100\%$. The mean percentage ($\bar{p}e$) of carry-over obtained from H1, H2, L1, L2 is $-0{\cdot}777\%$, with a standard error of mean (SEM) of 0·597%. Assuming normal distribution, the Student's t test results in $t=1{\cdot}30$. At $p=0{\cdot}05$ it is impossible to reject the null hypothesis that no difference exists in the mean percentage of carry-over and null percent (mean percentage of carry-over is not significant). The sequence L1, L2, H1, H2 results in $\bar{p}e=-0{\cdot}695\%$, SEM $=0{\cdot}749\%$, $t=0{\cdot}93$. In this situation the null hypothesis cannot be rejected either and the mean percentage of carry-over is not significant.

### *Comparison of methods experiment*

For the comparison of methods experiment (PSEP-4 [3]) the TDx therapeutic drug-monitoring system T-uptake results provides a measure of thyroxine binding sites in a sample. As a comparative method a T3-uptake assay, using a $^{125}$I RIA kit, was run. Due to the different methods of measuring T4-binding sites, results obtained with the T-uptake and T3-uptake are inversely related. To compare methods, the T-uptake values were mathematically transformed to percentage uptake values, according to Abbott [2], assuming a mean normal value of 29·5% (TDx transformed % uptake $=29{\cdot}5\%/\sqrt{0{\cdot}8\ (\text{T-uptake})^2+0{\cdot}2}$. One hundred patients' samples were analysed in duplicate for T-uptake and T3-uptake. The data points obtained were analysed statistically with the NCCLS accuracy computer program; no data points were excluded.

The statistical analysis included calculation of within-run imprecision for each method based on duplicate analysis, and an estimate of method accuracy from regression calculation. Table 2 presents estimates of imprecision, divided into three groups according to the results in percentage uptake. Mean values are shown as average concentrations of the group. These imprecision results obtained with patient sera for T-uptake are in agreement with the within-run imprecision results obtained by the replication experiment (see table 1).

The regression analysis between the T3-uptake ($x$ axis) and the T-uptake ($y$ axis) resulted in a slope of 0·881 (95% confidence range: 0·810 to 0·952) and an intercept of 5·082% (95% confidence range: 2·952% to 7·212%). The standard error of estimate, $S_{yx}$, is 2·52%.

It can be concluded, with respect to imprecision and accuracy, that the TDx therapeutic drug monitoring system gives good results for T-uptake. Taking in account the facts that the method is non-radioactive, homogeneous, applicable for stat analysis, calibration once a fortnight, the TDx is an attractive system for analysing T-uptake.

### References

1. National Committee for Clinical Laboratory Standards, Proposed Standard PSEP-3: *Protocol för Establishing Performance Claims for Clinical Chemical Methods. Replication Experiment* (NCCLS, USA, 1979).
2. Abbott Laboratories, Diagnostic Division, *TDx Operators Manual* (Abbott Laboratories, USA, 1983).
3. National Committee for Clinical Laboratory Standards Proposed Standard PSEP-4: *Protocol for Establishing Performance Claims for Clinical Methods. Comparison of Methods Experiment* (NCCLS, USA, 1979).

**INTERNATIONAL MEETING ON PRODUCT DESIGN ASSURANCE IN ENGINEERING**

*11–13 June 1985, Wembley Conference Centre, London*

The Society of Environmental Engineers' 1985 conference at Wembley will cover Specifications, Techniques and Case studies.

In conjunction with the conference there will be a major exhibition of environmental equipment.

*Details from Mrs Helen Gibbons, Society of Environmental Engineers Secretariat, Owles Hall, Buntingford, Hertfordshire, UK. Tel.: 0763 71209.*

**'BIORHYTHMS'**

*Sample Page*

**EVOLUTION**

Charlie Darwin based his thesis
On the Origin of Species
On his journeys for collection
On meticulous detection
So we've heard

And then claimed that his solution
To the mode of Evolution
Withstood rigorous inspection
And was Natural Selection
In a word

And that only fools and mystics
Could believe characteristics
That you might acquire in living
To your progeny you're giving
That's absurd

Basic biology has been set to words and music in a new series distributed for Learn through Music Ltd by Taylor & Francis Ltd. *Biorhythms* songs were written by Harold Baum (Professor of Biochemistry at Chelsea College, London) who produced the successful *Biochemist's Songbook.*

*Biorhythms 1—Human Biology* and *2—General Biology* each cost £8·95: the package contains one book and one audio-cassette.

*Orders to Taylor & Francis Ltd, Rankine Road, Basingstoke, Hampshire RG24 0PR, UK.*

